# Next generation sequencing of vitreoretinal lymphomas from small-volume intraocular liquid biopsies: new routes to targeted therapies

**DOI:** 10.18632/oncotarget.14008

**Published:** 2016-12-17

**Authors:** Andi K. Cani, Daniel H. Hovelson, Hakan Demirci, Mark W. Johnson, Scott A. Tomlins, Rajesh C. Rao

**Affiliations:** ^1^ Michigan Center for Translational Pathology, University of Michigan, Ann Arbor, MI, US; ^2^ Department of Pathology, University of Michigan, Ann Arbor, MI, US; ^3^ Department of Computational Medicine & Bioinformatics, University of Michigan, Ann Arbor, MI, US; ^4^ Department of Ophthalmology and Visual Sciences, W.K. Kellogg Eye Center, University of Michigan, Ann Arbor, MI, US; ^5^ Department of Urology, University of Michigan, Ann Arbor, MI, US; ^6^ Comprehensive Cancer Center, University of Michigan, Ann Arbor, MI, US; ^7^ A. Alfred Taubman Medical Research Institute, University of Michigan, Ann Arbor, MI, US; ^8^ Section of Ophthalmology, Surgical Service, Veterans Administration Ann Arbor, Healthcare System, Ann Arbor, MI, US

**Keywords:** next generation sequencing, vitreoretinal lymphoma, primary central nervous system lymphoma, biopsy, precision medicine

## Abstract

**Background:**

Vitreoretinal lymphoma (VRL), the most common lymphoma of the eye, is a rare form of primary CNS lymphoma (PCNSL). Most frequently a high-grade diffuse large B cell lymphoma, VRL can cause vision loss and its prognosis remains dismal: the overall survival time is 3 years after diagnosis. Radiotherapy and chemotherapy are used but remain frequently ineffective, and no standardized treatment regimen exists. Furthermore, no biologically targeted treatments, based on the genetic profile of the tumor, are available, as VRL has hitherto not comprehensively been profiled. To address these unmet needs, we hypothesized that a next generation sequencing (NGS)-based, National Cancer Institute (NCI) MATCH Trial-modified panel would be able to identify actionable genomic alterations from small-volume, intraocular liquid biopsies.

**Methods and Findings:**

In this retrospective study, we collected diluted vitreous biopsies from 4 patients with a high suspicion for VRL. Following cytological confirmation of lymphoma (all were diffuse large B cell lymphomas), we subjected genomic DNA from the biopsies to NGS, using a panel containing 126 genes (3,435 amplicons across several hotspots per gene), which was modified from that of the NCI MATCH Trial, a new trial that has matched patients with cancers that have not responded (or never responded), to investigational therapeutics based on their prioritized mutation profile rather than site of tumor origin. Using a validated bioinformatics pipeline, we assessed for the presence of actionable mutations and copy number alterations. In all four small-volume, intraocular liquid biopsies, we obtained sufficient genomic DNA for analysis, even in diluted samples in which the undiluted vitreous was used for cytology and flow cytometry. Using NGS, we found targetable heterozygous gain-of-function mutations in the *MYD88* oncogene, and confirmed in our cohort the presence the L265 mutations, previously described using PCR-based assays. For the first time in VRL, we also identified the *MYD88* S243N mutation. We also identified two-copy copy number losses in the tumor suppressor *CDKN2A* in all four cases, and one copy loss of the tumor suppressor *PTEN* in one sample. In one case, in which vitreous biopsies were originally read as cytologically negative, but which was confirmed as lymphoma when a lesion appeared in the brain two years later, our NGS-based approach detected tumoral DNA in the banked, original liquid biopsy.

**Conclusions:**

We performed the first systematic exploration of the actionable cancer genome in VRL. Our NGS-based approach identified exploitable genomic alterations such as gain-of-function *MYD88* oncogene mutations and loss of the tumor suppressor *CDKN2A*, and thus illuminates new routes to biologically targeted therapies for VRL, a cancer with a dismal prognosis. This precision medicine strategy could be used to nominate novel, targeted therapies in lymphomas and other blinding and deadly ocular, orbital, and ocular adnexal diseases for which few treatments exist.

## INTRODUCTION

Vitreoretinal lymphoma (VRL), the most common lymphoma of the eye, is linked closely to CNS lymphoma (PCNSL), and affects ~380 individuals in the U.S per year.[1] Fifty to 90% of those with primary VRL have concurrent (or will develop) PCNSL, while 15-25% of patients with primary PCNSL develop VRL.[1] Often mistaken for uveitis, delays in VRL diagnosis are common. Diagnosis relies on small-volume intraocular (vitreous) fluid biopsy, yet the limited number of malignant cells recovered complicates cytological analysis, especially in distinguishing from inflammatory lymphoid infiltrates. Flow cytometry, immunohistochemistry, and cytokine analyses are suggestive, but not diagnostic. The high viscosity of vitreous fluid often interferes with sampling and can confound cytological analyses and flow cytometry, which can lead to false-negatives, though this can be dependent on experience of consultant pathologists.[2, 3]

Most frequently a high-grade diffuse large B cell lymphoma (DLBCL), VRL can cause vision loss, with progression-free survival of ~1 yr and overall survival less than 3 years after diagnosis. Radiotherapy and chemotherapy are used but are frequently ineffective: no standardized treatments exist. Since VRLs have never been comprehensively profiled, no genetically targeted treatments exist. To address these unmet needs, we hypothesized that a next generation sequencing (NGS)-based panel, a modified version of that used in the National Cancer Institute-Molecular Analysis for Therapy Choice (NCI-MATCH) Trial (NCT02465060),[4, 5] would identify actionable genomic alterations from small-volume, intraocular liquid biopsies and nominate precision medicine-based treatment strategies. The modified NCI-MATCH panel we used herein is designed to detect actionable alterations in both advanced solid tumors *and lymphomas* (http://www.cancer.gov/about-cancer/treatment/clinical-trials/nci-supported/nci-match).[6] We analyzed four cytology-confirmed VRL cases, which represents about 1% of the VRL cases that occur in the U.S. annually (~380/yr).[1]

## RESULTS

**Figure 1:**
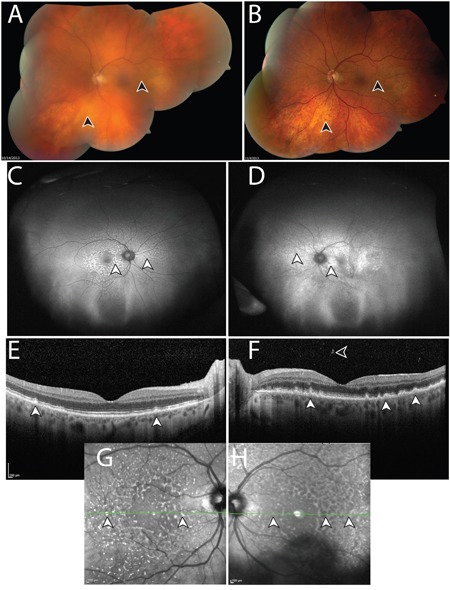
Manifestations of vitreoretinal lymphoma in Case 103 A. Montaged fundus photo of the left eye with vitreous debris prior to intraocular liquid biopsy and vitrectomy. Lymphoma cells are suspended in the vitreous, resulting in a “hazy” view, which obscures anatomic details of the retina (arrowheads). B. Following intraocular liquid biopsy and vitrectomy, which did not detect malignant cells, the media of the left eye is clear and retinal details can be discerned, such as subretinal lipofuscin clumps, and sub-retinal pigment epithelium (RPE) deposits, which manifest in a yellow and dark stippled, leopard-like pattern (arrowheads). Ultra-wide field fundus autofluorescence of the right C. and left D. eye, shows stippled hyper-autofluorescence corresponding to the lymphomatous sub-RPE deposits (arrowheads). Optical coherence tomography of the right E. and left F. eye shows nodular hyperreflective lymphomatous lesions at the RPE level (arrowheads). Prior to biopsy of the left eye (F), lymphoma cells can be seen in the posterior vitreous. Insets G, H. represent near infrared reflectance imaging of the right (G) and left (H) eyes, which highlight the leopard-like pattern of the sub-RPE lymphomatous macular infiltrates. Green lines and arrowheads of insets (G, H) correspond to the cross sectional plane of the OCT images in (E) and (F). Similar to (A), autofluorescence (D), OCT (F), and near infrared reflectance imaging (H) in the left eye appear blurry compared to the right eye due to the presence of lymphoma cells in the vitreous. Except for (B), images were obtained following biopsy and vitrectomy in the right eye (C, E, G) but prior to these interventions in the left eye (A, D, F, H). During this time, visual acuity was within normal range.

**Figure 2:**
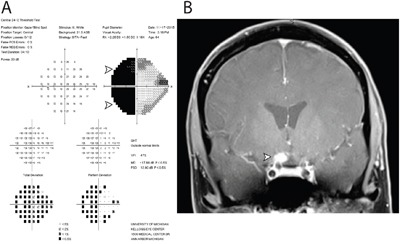
Functional and structural cause of vision loss in Case 103 Two years following negative cytology results from intraocular liquid biopsies in each eye, the patient developed severe vision loss in the left eye. A. Humphrey 24-2 visual field testing report of the left eye revealed a dense temporal hemianopia, denoted by black area (arrowheads), which signified lack of sensitivity to light stimulus in the temporal half of visual field. B. Magnetic resonance imaging with gadolinium contrast revealed an enhancing lesion abutting the right optic chiasm and optic nerve. Subsequent cytological analysis of cerebrospinal fluid confirmed the diagnosis of primary CNS lymphoma, diffuse large-B cell subtype.

We collected four small-volume vitreous biopsies from four patients with a high suspicion for VRL. All (cases 101-104) were males in their 60s. Cases 101 and 102 were diagnosed with PCNSL prior to biopsy, and showed cytology-proven VRL on vitreous biopsy (DLBCL). Cases 103 and 104 underwent vitreous biopsy in both eyes after developing vitreous debris and subretinal infiltrates bilaterally (Case 103, Figure 1A-1H), yet vitreous cytological analyses were negative. Two years later, Case 103 developed vision loss with right hemianopia (Figure 2A) and MRI (Figure 2B) revealed a lesion in the right optic nerve and chiasm. Since cytologic analysis of CSF fluid confirmed PCNSL (DLBCL), the patient's earlier ocular presentation was presumed bilateral VRL. Case 104 exhibited painless and chronic, bilateral vitreous debris for 2 years with a negative workup for uveitis or other systemic causes of inflammation. This patient also had a right parietal lobe lesion on MRI, suggestive of PCNSL with VRL.

**Figure 3:**
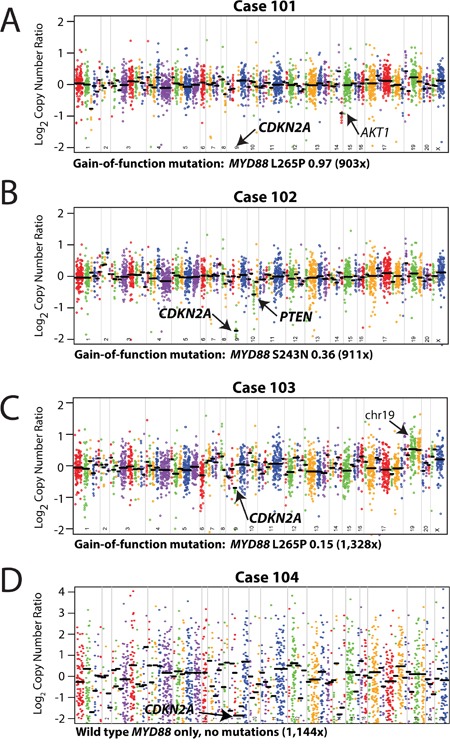
Mutation and copy-number analysis of vitreoretinal lymphomas from next generation sequencing data Copy-number profiles, with prioritized alterations in MYD88 (including point mutation/variant, variant fraction in the sample, and overall coverage depth [sum of the number of variant-containing reads and the number of reads without the variant]) are listed below each plot, for each of the three vitreoretinal lymphomas: A. Case 101, B. Case 102, C. Case 103, and D. Case 104. For each sequenced vitreoretinal lymphoma case, GC-content corrected, normalized read counts per amplicon were divided by those from a composite normal sample, yielding a tumor-to-normal copy-number ratio for each amplicon. Log2 tumor-to-normal copy-number ratios per amplicon are plotted (with each individual amplicon represented by a single dot, and each individual gene indicated by different colors), with gene-level copy-number estimates (black bars) determined by taking the weighted mean of the per-probe copy-number ratios. Prioritized high-level copy number alterations are indicated in bold. Log2 copy-number ratios for CDKN2A for Case 101 (A) are off the scale. Annotated, but not prioritized, one copy number losses in AKT1 (A, Case 101), and low level copy number gains in genes on chromosome (chr) 19 (C, Case 103) are noted by arrows.

Sufficient genomic DNA was recovered from each vitreous biopsy (Table 1). NGS was performed using the DNA component of the Oncomine Comprehensive Assay, a panel comprised of 3,435 amplicons targeting 126 genes, a modified version of which is being used in the NCI-MATCH trial,[6] and sequencing on the Ion Torrent Proton sequencer.[5] Targeted genes were selected based on pan-solid tumor NGS and copy number profiling data analysis to prioritize somatic, recurrently altered oncogenes, tumors suppressors and genes present in high level copy number alterations, filtered by available or investigational therapeutic targets.[4, 5] All four samples provided informative data, and sample statistics are listed in Table 1. We identified one non-synonymous point mutation each in Cases 101-103 and one high level deletion in each sample (Figure 3A-3D).

**Table 1:** Summary NGS Statistics

Case	DNA (ng)	Mapped Reads	On Target	Depth	Uniformity
101	412.5	2,636,783	98.86%	832.6	93.95%
102	247	2,778,318	99.06%	872.3	93.76%
103	5.8	3,548,338	99.04%	1,128	94.73%
104	26	4,377,192	98.90%	1,144	62.77%
**Average**	172.8	3,335,157	98.97%	994	86.30%
**SD**	193.6	801,889	0.10%	164	15.69%

Means and corresponding standard deviations (SD) are given for the amount of genomic DNA isolated from each intraocular liquid biopsy, number of mapped reads, on target reads, coverage depth, and uniformity in next generation sequencing summary table for each case.

Three of the four VRLs (75%) harbored prioritized gain-of-function mutations in the Toll/interleukin-1 receptor (TIR) domain of *MYD88*. MYD88 is an adaptor protein that binds to the intracellular domains of Toll-like receptors (TLRs) and interleukin-1 receptor on B-cells and macrophages, which stimulates NF-κB signaling pathway, and is involved in innate immunity.[7] These included two p.L265P (cases 101 homozygous and 103 heterozygous) and one p.S243N (case 102 heterozygous) mutations all at presumed clonal, variant frequency (Figure 3A-3C).

We also assessed somatic copy number alterations from the same NGS data using a validated approach,[8] with case-specific gene (and amplicon) level copy number plots (Figure 3A-3D). We identified few copy number alterations in our VRL samples, however we identified prioritized, high level copy loss of *CDKN2A* in all samples. High level copy loss was observed in cases 101, 102, and 104, while case 103 also harbored a high level loss given the presumed tumor content of 30% (based on *MYD88* L265P at 0.15 variant allele frequency). We also detected a one-copy deletion in *PTEN* in case 102. Lastly, although not prioritized, we identified a focal one-copy loss in *AKT1* in case 101 and low level gain of chromosome 19 was observed in case 103 (targeted genes included *STK1, MLL4/KMT2D, and ARHGAP43*).

## DISCUSSION

**Figure 4:**
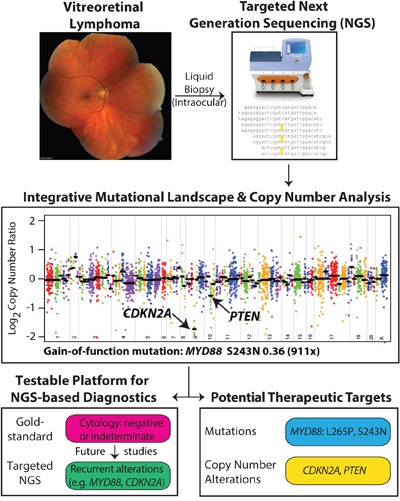
Workflow of determining driver and potentially actionable genomic alterations Genomic DNA from intraocular liquid biopsies is subjected to targeted next generation sequencing using a cancer gene panel. Bioinformatics analysis yields candidate point mutations and copy number alterations that potentially drive tumor growth and development in vitreoretinal lymphomas. Potentially actionable therapeutic targets are prioritized and reported.

We demonstrate the feasibility of targeted NGS on intraocular liquid biopsies using minute volumes (as little as 500 microliters) of non-diluted and diluted vitreous. Our approach does not compromise the volumes needed for cytology-based and other diagnostics (e.g. flow cytometry). Our NGS approach may overcome difficulties related to high vitreous viscosity, poor cellular preservation, low cellularity, false-negative and false-positive PCR-based results, which often leads to delays in diagnosis and therapy (Figure 4).[9] For instance, in case 103, presumably false-negative cytologic interpretations of biopsies in both eyes led to a two-year delay in therapy, when PCNSL cells were finally detected in the CSF. Despite repeated false-negative findings in Cases 103 and 104, our NGS strategy detected actionable genomic alterations in as little as 5.8 ng of tumor DNA from the original biopsy.

*MYD88* GOF mutations were found in 3 of 4 samples (75%) and high level copy number loss of *CDKN2A* were found in all 4 samples (100%). All *MYD88* mutations occurred in the TIR domain, and 2 of 4 samples (50%) harbored the p.L265P point mutation. We detected *MYD88* p.S243N mutation (1 of 4 samples, 25%), high level *CDKN2A* loss (4 of 4 samples, 100%), and low level *PTEN* loss (1 of 4 samples, 25%) for the first time in VRL.

Nearly all of these alterations are potentially targetable. For instance, currently recruiting clinical trials in DLBCLs assessing TLR inhibitors require the *MYD88* p.L265P mutation as an entry criterion (https://clinicaltrials.gov/ct2/results?term=myd88; accessed 8/8/16). Both p.L265P and p.S243N *MYD88* mutations show high levels of NF-κB transactivation, which can potentially be targeted by the Bruton's kinase inhibitor ibrutinib, and IRAK1/4 antagonists.[10–12] *MYD88* mutations other than p.L265P make up a quarter of all *MYD88* mutations in patients with DLBCLs and unlike previous limited studies that solely evaluate for the p.L265P mutation (and one study that reported p.P258L),[13–15] our comprehensive NGS-based approach reveals other potentially actionable, and diagnostic, GOF *MYD88* alterations in VRL.

High level *CDKN2A* loss is considered potentially targetable, as ilorasertib (an inhibitor of Aurora, VEGF, and PDGF tyrosine kinase families;https://clinicaltrials.gov/ct2/show/NCT02540876; accessed 8/8/16) and palbociclib (a CDK4/6 inhibitor;https://clinicaltrials.gov/ct2/show/NCT02693535; accessed 8/8/16) are being tested in advanced *CDKN2A*-deficient tumors. Likewise, although observed at only a single copy loss in our study, *PTEN*-deficient tumors are being targeted through PI3K-beta inhibition (https://clinicaltrials.gov/ct2/show/NCT02465060), as well as similar strategies targeting other components of the PTEN-PI3K-AKT pathway (e.g.https://clinicaltrials.gov/ct2/show/NCT02761694 and others).

Although not prioritized, an annotated one-copy loss in the oncogene *AKT1* was observed in case 101. While oncogenic *AKT1* GOF mutations and amplifications have been linked to a variety of cancers [16], a recent report has found recurrent one-copy *losses* of *AKT1* in DLBCLs [17]. How one-copy loss of a known oncogene like *AKT1* might be related to DLBCL/VRL tumorigenesis requires further investigation.

There are limitations in our study. While our analyzed samples represent > 1% of the number of VRLs that occur annually in the U.S., our sample size of 4 is modest. Future studies should investigate a larger cohort of VRL samples. While VRL is considered a subtype of PCNSL, similarities in their respective genetic landscapes have not yet been explicitly tested. A future investigation should focus on comparing the genetic alterations between PCNSL and VRL, especially in paired sample sets in patients with lymphoma cells in the eye (vitreous or choroid) and elsewhere in the CNS (cerebrospinal fluid, brain, spinal cord). Finally, while the modified, NCI-MATCH panel we employ is designed to detect alterations in solid tumors *and lymphoma*, a custom NGS panel enriched for potential non Hodgkin lymphoma targets may be able to capture alterations not covered in our panel.

As improved therapies are urgently needed for VRLs due to dismal survival rates, high rates of relapse as well as local and vision-related toxicities of present treatments (e.g. radiotherapy), our results demonstrate the utility of an NGS-based approach to nominate precision therapeutic approaches for VRLs. The route of administration of therapy employed would depend on how currently available targeted agents (FDA-approved for other cancers or in clinical trial) are delivered, and may be systemic (oral, intravenous, intrathecal, etc). Our NGS-based strategy is not intended to replace standard diagnostic methods, but to complement it. In cases in which VRL is confirmed by standard-of-care cytology based, diagnostic methods, our NGS-based strategy would be an *adjunctive* approach to characterize actionable targets and nominate potential therapeutic strategies. In our previous work, we confirmed that the filtering criteria employed here identify prioritized high-confidence somatic variants in tumoral DNA that pass Sanger sequencing validation, the main comparator technique, with >95% accuracy [4]. This approach could potentially be used for detecting intraocular recurrence and monitoring treatment response, which currently remains difficult. If our results are confirmed in larger cohort analyses and are employed as an adjunctive strategy for VRL, we recognize that NGS-based testing may not be feasible for every clinical site. We envision that after collection of undiluted vitreous for cytology and other point-of-care diagnostic tests, diluted samples could be flash frozen and sent to centers with expertise in genomic DNA collection from vitreous, NGS, and bioinformatics analyses.

To our knowledge, this is the first NGS study to comprehensively profile VRLs. This precision medicine approach may have potential diagnostic and therapeutic applications in characterizing small-volume, liquid biopsies in order to nominate rationally driven clinical trials for ocular diseases with few therapeutic options.

## MATERIALS AND METHODS

### Case selection

The study was carried out at the highest ethical standards and with the approval of the University of Michigan Institutional Review Board. We identified a cohort of three archived, flash-frozen vitreous specimens linked to biopsy-proven VRL or PCNSL from the intraocular liquid biobank (Cases 101-103) and one fresh specimen highly suspicious for VRL (Case 104) at the Kellogg Eye Center, Department of Ophthalmology & Visual Sciences at the University of Michigan for next generation sequencing (NGS). Clinicopathological information for each case was obtained from the clinical archive.

### Targeted next generation sequencing (NGS)

Targeted next generation sequencing was performed essentially as previously described, with few modifications relating to sample preparation [4, 5, 18, 19]. Each intraocular liquid specimen was thawed and centrifuged to pellet tumor cells. Genomic DNA was isolated using the Qiagen Allprep formalin-fixed paraffin-embedded DNA/RNA kit (Qiagen, Valencia, CA) and quantified as previously described. Targeted, multiplexed NGS was performed by Ion Torrent NGS using the DNA component of a beta version of the Oncomine Comprehensive Assay (OCP), a custom panel comprised of 3,435 amplicons targeting 126 genes. Genes included in this panel were selected based on pan-solid tumor next generation sequencing and copy number profiling data analysis that prioritized somatic, recurrently altered oncogenes, tumors suppressors, genes present in high level copy gains/losses and known/investigational therapeutic targets [4]. Library preparation with barcode incorporation, template preparation and sequencing using the Ion Torrent Proton sequencer were performed according to the manufacturer's instructions. Data analysis was performed using Torrent Suite 4.0.2, with alignment by TMAP using default parameters, and variant calling using the Torrent Variant Caller plugin (version 4.0-r76860) using default low-stringency somatic variant settings. Variant annotation filtering and prioritization was performed essentially as described using validated in house pipelines [4, 18–21]. Briefly, called variants were filtered to remove synonymous or non-coding variants, those with flow corrected read depths (FDP) less than 20, flow corrected variant allele containing reads (FAO) less than 6, variant allele frequencies (FAO/FDP) less than 0.10, extreme skewing of forward/reverse flow corrected reads calling the variant (FSAF/FSAR <0.2 or >5), or indels within homopolymer runs >4. Called variants were filtered using a panel-specific, in house blacklist. Variants with allele frequencies >0.5% in Exome Sequencing Project 6500 (ESP6500) or the 1000 Genomes project, and those reported in ESP6500 or 1000 genomes with observed variant fractions between 0.40 and 0.60 or > 0.9 were considered germline variants unless occurring at a known hot-spot variant. Variants located at the last mapped base (or outside) of amplicon target regions, variants with the majority of supporting reads harboring additional mismatches or indels (likely sequencing error), those in repeat–rich regions (likely mapping artifacts), and variants occurring exclusively in one amplicon if overlapping amplicons cover the variant were excluded. High confidence somatic variants passing the above criteria were then visually confirmed in Integrative Genomics Viewer (https://www.broadinstitute.org/igv/). We have previously confirmed that these filtering criteria identify prioritized high-confidence somatic variants that pass Sanger sequencing validation with >95% accuracy [18–22]. Copy number analysis from total amplicon read counts provided by the Coverage Analysis Plug-in (v4.0-r77897) was performed essentially as described using a validated approach [4, 5, 8, 18, 19]. Genes with a log_2_ copy number estimate of <-1 or >0.6 were considered to have high level loss or gain, respectively.

To prioritize potential driving alterations, we utilized Oncomine software tools (https://powertools.oncomine.com) to annotate called variants, which use pan-cancer next generation sequencing data to identify genes as oncogenes or tumor suppressors, based on overrepresentation of hot-spot or deleterious mutations, respectively. Variants in oncogenes are then considered gain-of-function if at a hot-spot and variants in tumor suppressors are considered loss-of-function if deleterious or at a hot-spot [4, 18]. Likewise, high-level copy number alterations were prioritized if they were concordant with the minimal common region analysis used to design the Oncomine Comprehensive Assay (e.g. high level copy number gain in a gene prioritized as amplified/deleted by minimal common region analysis.
